# Pharmacogenomics of CYP2C9: Functional and Clinical Considerations^†^

**DOI:** 10.3390/jpm8010001

**Published:** 2017-12-28

**Authors:** Ann K. Daly, Allan E. Rettie, Douglas M. Fowler, John O. Miners

**Affiliations:** 1 Institute of Cellular Medicine, Newcastle University, Framlington Place, Newcastle upon Tyne NE2 4HH, UK; 2 Department of Medicinal Chemistry, University of Washington, Seattle, WA 98195, USA; rettie@uw.edu; 3 Department of Genome Sciences and Department of Bioengineering, University of Washington, Seattle, WA 98195, USA; dfowler@uw.edu; 4 Department of Clinical Pharmacology, Flinders University School of Medicine, Adelaide 5042, Australia; john.miners@flinders.edu.au

**Keywords:** CYP2C9, cytochrome P450, polymorphism, pharmacogenomics, warfarin

## Abstract

CYP2C9 is the most abundant CYP2C subfamily enzyme in human liver and the most important contributor from this subfamily to drug metabolism. Polymorphisms resulting in decreased enzyme activity are common in the *CYP2C9* gene and this, combined with narrow therapeutic indices for several key drug substrates, results in some important issues relating to drug safety and efficacy. CYP2C9 substrate selectivity is detailed and, based on crystal structures for the enzyme, we describe how CYP2C9 catalyzes these reactions. Factors relevant to clinical response to CYP2C9 substrates including inhibition, induction and genetic polymorphism are discussed in detail. In particular, we consider the issue of ethnic variation in pattern and frequency of genetic polymorphisms and clinical implications. Warfarin is the most well studied CYP2C9 substrate; recent work on use of dosing algorithms that include CYP2C9 genotype to improve patient safety during initiation of warfarin dosing are reviewed and prospects for their clinical implementation considered. Finally, we discuss a novel approach to cataloging the functional capabilities of rare ‘variants of uncertain significance’, which are increasingly detected as more exome and genome sequencing of diverse populations is conducted.

## 1. Introduction

The cytochrome P450 2C (CYP2C) subfamily comprises four enzymes: CYP2C8, CYP2C9, CYP2C18 and CYP2C19. Of these, CYP2C9 is the most abundantly expressed and contributes to drug metabolism to the greatest extent. Indeed, CYP2C9 accounts for approximately 20% of total hepatic P450 protein, based on mass spectrometry quantitation [1]. Significant expression additionally occurs in the gastrointestinal tract [2]. After CYP3A4 and CYP2D6, CYP2C9 is the next most important cytochrome P450 in terms of the numbers of therapeutic agents oxidized, contributing to the metabolism of approximately 15% of all drugs that are subject to P450-catalyzed biotransformation [3]. Importantly, as discussed below in Section 2 and Section 4, CYP2C9 is the major enzyme responsible for the metabolic clearance of several clinically used drugs that have a narrow therapeutic index. Thus, inter-individual variability in CYP2C9 protein expression and activity may impact the efficacy and safety of drug treatment. In this regard, the CYP2C9 protein content of human liver microsomes (HLM) varies by an order of magnitude [1] and activity in vivo, measured as the tolbutamide urinary metabolic ratio, was similarly found to vary by an order of magnitude in a group of healthy subjects that excluded poor metabolizers [4]. The occurrence of drug–drug interactions (DDIs), arising from inhibition or induction of CYP2C9, and genetic polymorphism further increase the extent of population variability in enzyme activity in vivo [5,6,7,8]. Here, we review aspects of CYP2C9 with particular reference to structure–function relationships and pharmacogenomics. Additional information on this subject area is available from several other recent review articles on various P450s that include coverage of CYP2C9 [9,10,11].

## 2. CYP2C9 Substrate Selectivity

The identification of CYP enzyme selective substrate and inhibitor ‘probes’ along with the availability of heterologously expressed recombinant human CYP enzymes that occurred over the last three decades has facilitated the development of reaction phenotyping procedures for characterizing the contribution of specific enzymes to a metabolic pathway. Of the various reaction phenotyping approaches adopted [12], the use of enzyme-specific inhibitors provides the least ambiguous means of characterizing the contribution of a CYP enzyme(s) to a metabolic pathway when HLM and hepatocytes are employed as the enzyme source. Identification of sulfaphenazole as a highly selective CYP2C9 inhibitor [13,14,15] has proved invaluable for determining the contribution of this enzyme to the metabolism of any given compound [5].

CYP2C9 contributes to the oxidation of a large number of drugs and also metabolizes a number of endogenous compounds, for example arachidonic acid, linoleic acid, and non-drug xenobiotics (e.g., galangin, methiocarb, pyrene, safrole, sulprofos and Δ-9-tetrahydrocannabinol). The range of substrates and their structures has been reviewed in detail previously [5,7,16,17]. The majority of substrates are weakly acidic compounds, although CYP2C9 also catalyzes the *N*-demethylation of a number of basic drugs (e.g., amitriptyline, fluoxetine and zopiclone).

Table 1 shows representative examples of drugs for which CYP2C9 is responsible for >25% of metabolic clearance. Sulfonylurea oral hypoglycemic agents, non-steroidal anti-inflammatory drugs (NSAIDs), and coumarin anticoagulants feature prominently. However, CYP2C9 contributes significantly to the metabolic clearance of drugs from other therapeutic classes, including the widely used anticonvulsant phenytoin [18,19], the diuretic torsemide [20] and the antihypertensive losartan [21]. In the latter case, CYP2C9 is the primary enzyme responsible for the conversion of losartan to its pharmacologically active metabolite E-3174. 

Several of the drugs listed in Table 1 have been employed as substrate ‘probes’ to measure CYP2C9 activity in vitro and in vivo. Diclofenac 4′-hydroxylation, *S*-flurbiprofen 4′-hydroxylation, losartan carboxylation, phenytoin 4′-hydroxylation, tolbutamide and torsemide tolylmethyl hydroxylation, and *S*-warfarin 7-hydroxylation activities have been used to assess CYP2C9 activity in vitro, with HLM, human hepatocytes and recombinant protein as enzyme sources, and as probe substrates in studies investigating drug and chemical inhibition of CYP2C9 and the influence of genetic polymorphism on CYP2C9 activity [5,6,22]. Each has its advantages and disadvantages, although tolbutamide and the structurally related torsemide have been proposed as a convenient compromise [13,23]. Most of the compounds utilized as substrates in vitro have similarly been employed, either individually or as part of a ‘cocktail’ of CYP enzyme substrate probes, to assess factors (genetic polymorphism, drug–drug interactions, disease states) that affect CYP2C9 activity in vivo [6,24]. An assessment of the drugs used as part of a ‘cocktail’ to assess CYP2C9 activity in vivo (diclofenac, flurbiprofen, losartan, tolbutamide and warfarin) recommended tolbutamide [24], although this drug is no longer available for clinical use in several countries. Recent studies on cocktails suitable for phenotype determination in resource-limited regions proposed losartan as the CYP2C9 activity probe [25], though a systematic evaluation of this approach similar to that performed previously [24] is still needed. A practical, though often overlooked, alternative is to use warfarin as the in vivo probe, but to administer vitamin K concomitantly to minimize safety concerns [26].

## 3. CYP2C9 Structure–Function

**Figure 1:**
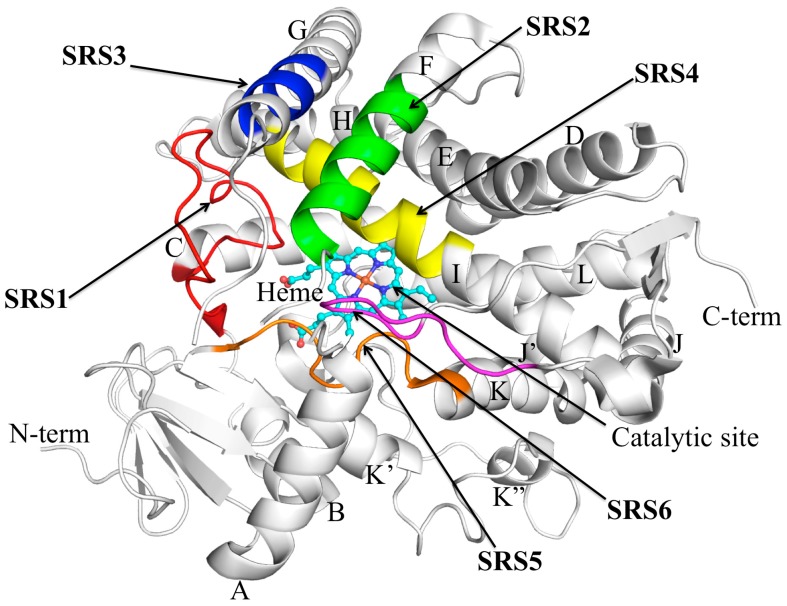
CYP2C9 structure showing substrate recognition sites (SRS) according to Gotoh [28]. The SRS are denoted by arrows: SRS1 (red), SRS2 (green), SRS3 (blue), SRS4 (yellow), SRS5 (orange) and SRS6 (magenta). The secondary structure elements of the rest of the protein are shown in white. The haem molecule is shown in ball and sticks with C, O, N, and Fe atoms in cyan, red, blue, and orange, respectively (from [29]). Reproduced with permission from Nair, P.C.; McKinnon, R.A.; Miners, J.O. Cytochrome P450 structure–function: insights from molecular dynamics simulations. *Drug Metab. Rev.* **2016**, *48*, 434–452; published by Taylor and Francis, 2016.

Knowledge of CYP2C9 structure–function relationships is well advanced due to the availability of X-ray crystal structures along with data from site-directed mutagenesis studies. Figure 1 provides an overview of the CYP2C9 structure with substrate recognition sites (SRS) indicated. Notably, an X-ray crystal structure of CYP2C9 complexed with flurbiprofen (Protein Data Bank code 1R90) in a catalytically favorable orientation has provided important insights into the binding of acidic substrates [27]. Specifically, this structure demonstrates a pivotal role for Arg108 in the binding of flurbiprofen. The conformational flexibility of the helix B to helix C loop region (see below) allows Arg108 to form a salt-bridge with the carboxylate group of flurbiprofen and additionally hydrogen bond with Asn289 and Asp293 on helix I, which serves to stabilize the conformation of Arg108 within the active site. Flurbiprofen packs into a hydrophobic cleft adjacent to helix-I that is formed by specific residues in SRS 1 (Val113, Phe114), 2 (Leu208), 3 (Val237, Met240), 4 (Gly296, Ala297), and 5 (Leu366).

Site-directed mutagenesis data are consistent with a pivotal role for Arg108 in the binding of acidic substrates. Substitution of Arg108 with Ala, Glu or Phe greatly reduced or abolished the metabolism of diclofenac and *S*-warfarin, whereas the Arg108Glu mutation had a negligible effect on the 1-hydroxylation of the unsubstituted polycyclic aromatic hydrocarbon pyrene and the 4-hydroxlation and dealkylation of propranolol (a basic compound) [30,31,32]. Confirmation of the roles of Val113 and Phe114 in stabilizing substrate binding via π–π interactions was demonstrated by reduced or complete loss of *S*-warfarin and diclofenac metabolism following substitution with Leu or Ile [33,34]. Lesser inhibition was observed with lauric acid, which lacks an aromatic group. Finally, mutagenesis of Asp293 to Ala, Asn or Val decreased activity towards dextromethorphan, diclofenac, pyrene, tolbutamide and S-warfarin in a substrate-independent manner due to reduced protein stability rather than an effect on substrate binding [32,35]. However, conservative replacement of Asp293 with Glu had only a minor effect on substrate binding and turnover [35].

In addition to the 1R90 structure described above, CYP2C9 X-ray crystal structures, with and without bound warfarin (PDB codes 1OG5 and 1OG2), have been solved [36]. Interestingly, in the 10G2 structure, warfarin was bound in the distal end of the active site cavity, possibly in an access channel [37], in an unproductive position some 10 Å from the heme iron. However, the constructs used to determine these structures were more extensively modified compared to that used to generate 1R90 [27]. In contrast to 1R90, Arg108 was oriented away from the active site in these structures. The non-involvement of Arg108 in the binding of ‘compound **1**’, a sulfone derivative from a drug discovery project, has similarly been reported for the X-ray crystal structure of CYP2C9 complexed with compound **1** (PDB code 4NZ2) [38]. As speculated by the authors of the latter study, conformational differences observed between the various CYP2C9 structures may arise from the mutations engineered into the proteins to facilitate crystallization, or possibly represent a dynamic equilibrium. 

In the latter regard, it is now well recognized that conformational flexibility (plasticity) is a feature of P450 proteins. Comparison of X-ray crystal structures reported for some CYP enzymes with different bound ligands together with molecular dynamics (MD) simulations demonstrate that dramatic ligand-induced conformational changes may follow substrate or inhibitor binding [29]. The transition from an ‘open’ to ‘closed’ state that occurs upon ligand binding may result in changes in the active site volume, the spatial positioning of backbone amino acids, and amino acid side-chain conformation. Notably, MD simulations of flurbiprofen-bound CYP2C9 showed the B–C loop region located approximately 20 Å away from the heme compared to the conformation adopted by the unliganded structure [39]. Given the inherent flexibility of CYP2C9 and other P450 proteins, different chemical classes of substrates and inhibitors may adopt different binding modes within the active site. For example, pharmacophore modeling data are consistent with distinct binding modes for acidic and ‘atypical’ basic (amine-containing) CYP2C9 substrates [16]. Thus, predicting the effects of genetic polymorphism on substrate and inhibitor binding (and hence kinetics) may not be straightforward using static X-ray crystal structures, particularly when the mutation is located outside of the active site (see Section 5.3 for further discussion).

## 4. Clinical Relevance of CYP2C9

### 4.1. Clinically Relevant Substrates

The emphasis in this section is on key CYP2C9 substrates used therapeutically, especially those where drug interactions and effects of genetic polymorphisms may affect treatment outcomes. Important substrates in terms of narrow therapeutic index and predominant metabolism by CYP2C9 are the coumarin anticoagulants, especially warfarin, and acenocoumarol, most sulfonylureas and phenytoin (Table 1).

Coumarin anticoagulants including warfarin are among the most widely prescribed drugs worldwide and are used to prevent thromboembolism in at risk individuals. Warfarin is the most widely used member of this class, but two other compounds with coumarin structures, acenocoumarol and phenoprocoumon, are preferred in some countries. All three drugs target the enzyme vitamin K epoxide reductase and require individualised dosing that is achieved by optimization of the coagulation rate (target International Normalized Ratio of 2–3 in most cases) following the start of treatment with titration of dose to achieve the required endpoint. The need to titrate dose arises in part due to interindividual variability in metabolism. An important role for CYP2C9 in the metabolism of the more active warfarin enantiomer, *S*-warfarin, is well established [40]. Though other P450s contribute to both *R*- and *S*-warfarin metabolism, data from studies concerned with drug–drug interactions and pharmacogenetics suggest that CYP2C9 is the key determinant of metabolism of this drug in vivo [41]. Acenocoumarol is also mainly metabolised by CYP2C9 [42], but there are some differences to warfarin with respect to rate of metabolism and enantiomer selectivity [43]. Phenprocoumon is also a CYP2C9 substrate but there appears to be a larger contribution to its oxidative metabolism from CYP3A4 than for the other two coumarin anticoagulants [44]. For the coumarin anticoagulants, there is a risk of serious bleeding if plasma drug concentrations are too high due, for example, to a drug–drug interaction or genetic polymorphism. Frequent measurement of international normalized ratio (INR) and dose adjustment mitigates the risk. Increasingly, direct acting oral anticoagulants such as dabigatran and rivoroxaban are being used in place of coumarin anticoagulants [45]. These are entirely different drugs to the coumarin anticoagulants with respect to their mechanism of action and there is no role for CYP2C9 in their metabolism.

Many of the older studies on CYP2C9 used tolbutamide as a model compound [5]. However, this first-generation sulfonylurea is now rarely prescribed, with second-generation compounds used widely instead for the treatment of type II diabetes [46]. These latter compounds are structurally related to tolbutamide but show some differences with respect to P450-mediated metabolism. The most widely used second-generation sulfonylurea in a number of different countries is gliclazide [47], which appears to be predominantly metabolized by CYP2C9. However, CYP2C19 also has at least a minor role in the metabolism of this compound [48], and some data suggest that this is also the case for glipizide [49]. As reviewed recently [50], with all the sulfonylureas, there is risk of hypoglycaemia if plasma drug concentrations are not maintained within the normal therapeutic range, and low CYP2C9 activity due to enzyme inhibition or genetic polymorphism may increase this risk.

On the basis of a similar chemical inhibitor sensitivity pattern to that for warfarin and tolbutamide, hydroxylation of phenytoin to p-HPPH was also demonstrated to be CYP2C9-mediated [51]. As with some of the sulfonylureas referred to above, there is also a minor contribution from CYP2C19. Phenytoin has a narrow therapeutic index and toxicity is associated with a variety of neurological symptoms; high plasma concentrations may additionally contribute to hepatic and skin toxicity reactions.

NSAIDs including diclofenac, flurbiprofen and ibuprofen are also well established CYP2C9 substrates. However, this group of drugs undergoes metabolism by other CYP enzymes including CYP2C8 and CYP3A4, often to different metabolites to those formed by CYP2C9, together with direct glucuronidation. For some other drugs including valproic acid and bosentan, metabolism by CYP2C9 appears important to treatment outcome under certain circumstances and this will be considered below in more detail. The angiotensin II receptor antagonist losartan is another interesting CYP2C9 substrate. An active carboxylic acid metabolite E-3174 is generated by CYP2C9 oxidation of the alcohol moiety. E-3174 has higher antagonist activity and a longer half-life than the parent drug [21]. Certain other sartan family members namely ibresartan [52] and, to a more limited extent candesartan [53] and valsartan [54], are also CYP2C9 substrates, but active metabolites are not generated. Other sartan family members are not metabolized by P450 [55]. In addition to prescribed drugs, CYP2C9 contributes to arachidonic acid metabolism with a role in the formation of epoxyeicosatrienoic acids (EETs) [56]. This pathway, which is not unique to CYP2C9, has nevertheless prompted a number of studies in relation to CYP2C9 genotype as a risk factor for several diseases (see Section 6.5).

### 4.2. CYP2C9 Inducers and Inhibitors

CYP2C9 is subject to inhibition by a wide range of drugs, both in vitro and in vivo [5,6]. Some of these are general P450 inhibitors such as cimetidine and ketoconazole and will not be considered in detail. However, there are a range of more specific and potent inhibitors that are useful in laboratory-based investigations and clinical trials, particularly sulfaphenazole which, as noted previously, is used to establish a role for CYP2C9 in metabolism in vitro. Other inhibitors include fluconazole, voriconazole and amiodarone. Amiodarone is often prescribed in combination with warfarin due to its antiarrhythmic effects. The DDI risk of this combination is well recognized [57], necessitating a change in warfarin dose of 6–65% [58]. The narrow therapeutic index of a range of widely prescribed CYP2C9 substrates discussed above means that there is a risk of clinically significant DDIs when these drug substrates and inhibitors are combined.

CYP2C9 is inducible by mechanisms involving various nuclear receptors including PXR, CAR, the glucocorticoid receptor, the estrogen receptor and the vitamin D receptor. Evidence for this range of induction mechanisms is provided by descriptions of some important DDIs involving induction, the existence of a variety of nuclear receptor response elements in the CYP2C9 promoter region and data from a range of in vitro studies on both primary hepatocytes and cell lines such as HepG2. Induction of CYP2C9 expression by individual ligands may involve more than one of the nuclear receptors and therefore several different promoter binding sites.

PXR is the best characterized nuclear receptor in terms of a role in CYP2C9 induction. In particular, clinical reports of rifampicin interactions with drugs now known to be CYP2C9 substrates emerged in the 1970s [59,60]. Rifampicin is a well-established ligand for human PXR [61,62] and other ligands identified soon after discovery of PXR include hyperforin, clotrimazole and nifedipine [61]. Phenobarbital is usually considered to interact with genes via another nuclear receptor (CAR), but it also binds to PXR and appears to be able to induce P450 expression via this receptor [63]. The CYP2C9 upstream region includes several CAR response elements and it was thought originally that CAR-related induction of CYP2C9 might be more important than PXR-related induction [64,65]. However, it now appears that CAR-specific inducers such as CITCO have a limited effect on CYP2C9 expression and that phenobarbital induction of CYP2C9 may involve mainly PXR [66]. On the basis of studies using both primary hepatocytes and in silico predictions, a wide range of structurally different drugs are known to bind to PXR and induce CYP3A4 activity including certain penicillins, cephalosporins and macrolides [67,68]. Similar studies specifically on CYP2C9 induction are more limited but are in general agreement with those for CYP3A4 [69]. Predictions that antimicrobials such as dicloxacillin and flucloxacillin are PXR inducers relevant to CYP2C9 expression are in line with data on drug–drug interactions for warfarin [70,71]. 

The estrogen receptor also appears to be a regulator of CYP2C9 expression, but in this case estradiol and ethinylestradiol interact with the receptor and decrease gene expression with antiestrogens including tamoxifen increasing expression [72]. This slightly unexpected finding is consistent with clinical data on metabolism of the CYP2C9 substrate losartan where women taking oral contraceptives show slower metabolism than other women [73].

## 5. *CYP2C9* Genetic Polymorphisms

### 5.1. Background

Evidence for the existence of a polymorphism affecting metabolism of the anti-diabetic drug tolbutamide was first reported in 1978 [74], but at that time it was thought that this might be related to the debrisoquine (CYP2D6) polymorphism. Subsequent studies showed that genetic regulation of tolbutamide metabolism was distinct from debrisoquine metabolism [15]. In parallel with these studies, an enzyme that could oxidize tolbutamide was cloned and later named CYP2C9 [75,76]. Analysis of CYP2C9 cDNA sequences provided evidence for the presence of two coding region polymorphisms resulting in the amino acid substitutions Arg144Cys and Ile359 Leu [77,78,79]. In vitro expression studies suggested these changes were functionally significant [75]. Genotyping of patients undergoing treatment with warfarin confirmed the functional importance of these polymorphisms [80,81,82]. The two polymorphisms (rs1799853 and rs1057910) form part of the CYP2C9*2 and CYP2C9*3 alleles. Both variants are found at relatively high frequencies in white Europeans and this, together with their well-established functional effects, has meant that they have been studied extensively in relation to metabolism of a wide range of drugs. However, they are not the only clinically relevant polymorphisms, and not even the most common variants in some ethnic groups.

### 5.2. Missense and Frameshift Variants in CYP2C9

CYP2C9*2 and *3 remain the best studied CYP2C9 alleles and are the most common variants based on combined global allele frequencies available to date [83]. A large number of generally less common alleles have also been identified; however, there is increasing data available about frequencies in populations worldwide from ongoing exome and genome sequencing efforts. When coding sequence polymorphisms are considered, the Exome Aggregation Consortium project (ExAC) (http://exac.broadinstitute.org/) [84] provides comprehensive data on frequencies in a range of populations for a large number of CYP2C9 single nucleotide variants (SNVs). There are limitations to these data including the fact that over 50% of samples sequenced are of European ethnicity, although African/African Americans, South Asians and East Asians are well represented here compared with other data sources or small published population surveys. A summary of all CYP2C9 variants associated with missense and frameshift mutations at “worldwide” frequencies down to 0.0001 based on the ExAC data is provided in Table 2. 

The summary of population frequencies in Table 2 shows the generally high frequencies of both CYP2C9*2 and *3 compared with other alleles though with some important interethnic differences. CYP2C9*3 is particularly common among South Asians and CYP2C9*2 is very rare among East Asians. While other variants associated with missense and frameshift mutations tend to be rare, CYP2C9*8 and CYP2C9*9 are more common than CYP2C9*2 and CYP2C9*3 in the African ethnicity group. Similarly, and in line with published reports, CYP2C9*11 is approximately 10 times more common among Africans compared with Europeans [85] though this allele and CYP2C9*12 appear to be the most common European alleles after CYP2C9*2 and *3 [86,87,88]. As shown in Table 2 and elsewhere [89], East Asians are very rarely positive for CYP2C9*2 and CYP2C9*3 is the most common variant allele. In particular, this ethnic group is positive for a range of rare missense alleles with CYP2C9*52 the most common after CYP2C9*3 [89]. South Indians are often positive for CYP2C9*2 and CYP2C9*3 but also show an appreciable frequency of CYP2C9*14 (0.02). This allele is much rarer in other populations.

Frameshift variants are very rare in *CYP2C9*, but one variant CYP2C9*6 with a single base pair deletion in exon 5 has been reported [95] (see Table 2). This variant will result in an inactive truncated protein, and is seen at a frequency of approx. 0.01 in Africans and more rarely in Hispanic-Latinos, but is very rare in other ethnicities. 

Sequencing studies on *CYP2C9* in some isolated populations have also been performed. For example, in a study of American Indian and Alaska native people, CYP2C9*2 and *3 were found at lower levels among the Yupik native people compared with other Alaskan residents, whereas the East Asian CYP2C9*29 alleles showed a frequency of 0.02 in the Yupiks with two novel missense variants, Met1Leu and Asn218Ile, seen at frequencies of 0.06 and 0.04 respectively [98]. It seems likely that further novel alleles will be detected as additional population sequencing studies of this type are performed.

Though the resequencing approach described above is the most informative as a means of studying genotypes in isolated populations where novel alleles may be important contributors to phenotype, there are also additional reports on the frequency of previously described polymorphisms in a number of such populations. For example, CYP2C9*3 was reported to occur at an unusually high frequency of 0.36 in a Malaysian aboriginal population, though CYP2C9*2, *4 and *5 were not detected [99]. In a survey of eight separate indigenous Mexican populations genotyped for CYP2C9*2, *3 and *6, CYP2C9*2 was detected at low frequencies in two tribes at higher frequencies than those reported for East Asians and CYP2C9*3 in six tribes with frequencies varying between 0.037 and 0.104 [100]. These findings are consistent with Asian-European admixture in these groups. Frequencies of known *CYP2C9* alleles in other indigenous North American populations are reviewed in detail in another article in this issue [101]. There is also a recent review of frequencies in a range of populations worldwide [102].

### 5.3. Functional Significance of CYP2C9 Missense Variants

The functional significance of many of the CYP2C9 variants listed in Table 2 has been investigated widely, especially in the case of the common variants. The availability of detailed structural information on CYP2C9 enables in silico prediction of the effect of amino acid changes from sequencing data. However, only one of the variants shown in Table 2 (CYP2C9*52) results in mutation of a residue of known to contribute to substrate binding (viz. Thr299Ala), based on the X-ray crystal structures of flurbiprofen- and warfarin-bound CYP2C9 [96]. Several other rare variants (*28, *30, *52, *55, *57) listed in the CYP alleles database (but not in Table 2) also fall within the active site, although no activity data are available for the last three. The ExAC database provides predictions based on Sorting Intolerant From Tolerant (SIFT), which makes predictions primarily from evolutionary sequence data together with known effects of amino acid changes and PolyPhen, which uses both sequence homology and structural predictions. These indices are useful but imperfect predictors of effect and do not necessarily correlate completely [103], as discussed in detail elsewhere for CYP2C9 variants [104]. As summarized in the effect column (Table 2), the two indices also do not correlate well in terms of predicting effects of missense mutations on CYP2C9 activity. In addition to these in silico predictions, data on variant effects are also available from in vitro expression studies and/or phenotypic studies in vivo involving either pharmacokinetic measurements or pharmacodynamic endpoints such as warfarin dose requirement. 

While it is acknowledged that binding residues may well differ between substrates (especially those from different chemical classes), the molecular basis of the reduced activity observed for most variants is not immediately apparent. Although there is evidence from in vitro studies to suggest that the magnitude of the reduction in enzyme activity associated with some variants (e.g., CYP2C9*2) may be substrate dependent (e.g., [22,93]), comparisons are not straightforward especially when different enzyme sources are employed to generate kinetic data. It has been demonstrated that *K_m_* values (and hence intrinsic clearances) for CYP2C9 substrates can vary between enzyme sources (e.g., liver microsomes, recombinant CYP2C9 expressed in different cell lines) due to the inhibitory effects of unsaturated long-chain fatty acids released during the course of an incubation [105]. Thus, while comparison of data obtained for variant CYP2C9 enzymes expressed in the same cell line is expected to be valid, caution is necessary in the interpretation of data obtained for variants expressed in different cell lines. 

Almost all the relatively common variants listed in Table 2 result in decreased activity based on a combination of in silico and in vitro or in vivo evidence. However, for the variants associated with the CYP2C9*2, *8 and *9 alleles, the situation is less clear. For CYP2C9*2, it was proposed initially that the Arg144Cys substitution alters the interaction with the electron donor cytochrome P450 oxidoreductase, thereby reducing catalytic efficiency [106]. However, subsequent data indicated that alterations in the P450 catalytic cycle, due to different degrees of coupling and uncoupling, were more likely responsible for the lower activities of CYP2C9*2 and also CYP2C9*3 [107]. Structural changes in the CYP2C9*2 and CYP2C9*3 proteins have been investigated using MD simulation [108,109,110]. The simulations variously predicted that expansion of the active site volume (with reduced probability of substrate binding in a catalytically favorable pose), increased interatomic distance between the site of metabolism and the oxyferryl heme center, reduction in the size of the substrate entry channel, and impaired hydrogen bonding with stabilizing amino acids contribute to the reduced activity of CYP2C9*2 and CYP2C9*3. Although ligand-dependent conformational changes in CYP proteins are well accepted, differences in the computational approaches adopted undoubtedly contribute to the different results observed in the three studies [29]. For CYP2C9*3, the data are more consistent with almost all studies investigating the activity of CYP2C9 variants finding that the Ile359Leu substitution associated with the CYP2C9*3 allele typically results in a greater than 80% reduction in the in vitro intrinsic clearances of CYP2C9 substrates. A recent X-ray crystal structure of CYP2C9*3 with losartan bound suggests that the effect of the Ile359Leu substitution, located a distant 15 Å from the active site, is transduced to neighboring I-helix residues that secondarily influence the β4 loop, which is important for substrate interactions near the heme [37].

The activity of the CYP2C9*8-encoded enzyme is also relatively poorly understood. PolyPhen and SIFT predict no effect of the Arg150His change on enzyme activity. In vivo studies on warfarin and phenytoin clearance suggest decreased activity [90,92]. In vitro studies are limited but there is a report of increased clearance and one of a 30% decrease in warfarin clearance [91,93]. The presence of upstream polymorphisms in linkage disequilibrium with the coding variant could explain these discrepancies and is discussed in detail below (Section 5.4).

CYP2C9*9 is predicted to be associated with decreased enzyme activity by both SIFT and PolyPhen. Data on the variant is limited but no effect on phenytoin clearance in vivo was found [90] and the available in vitro expression studies report slight decreases in warfarin and tolbutamide intrinsic clearance that were not statistically significant [91,93]. 

Overall, as discussed above and summarized in Table 2, there is convincing evidence for decreased activity from the CYP2C9*3, CYP2C9*5, CYP2C9*6, CYP2C9*11, CYP2C9*12 and CYP2C9*13 gene products, and the CYP2C9*2 protein is also associated with impaired metabolism with at least some substrates. However, evidence that other alleles listed in Table 2 such as CYP2C9*14, CYP2C9*27, and CYP2C9*29 code for proteins that show functionally significant decreases in activity is based only on in vitro studies in bacteria, insect cells and/or COS-7 cells [89,93,111]. The use of several substrates that show good correlations in activity for the different variants in the same expression system is a positive [93], but further evaluation of the relevance of these variants in vivo, especially in relation to warfarin dosing, would be useful.

### 5.4. Variants in Non-Coding Regions

The ExAC project is mainly concerned with coding region variants but does provide some data on non-coding variation in regions close to exons. However, all variants reported for CYP2C9 in these regions are very rare (<0.0001) in each study population. There have been a number of studies of polymorphism in upstream sequences covering the region up to approx. 10,000 bp upstream of the translation start site. Overall, these indicate that coding region missense polymorphisms are a more important contributor to inter-individual variation in CYP2C9 activity, but a few interesting findings in relation to the non-coding variants have also emerged. 

Upstream region variants and their functional significance are most easily considered in CYP2C9 haplotypes where there are no coding region polymorphisms present. A C>T variant at −1188 (rs4918758) has been reported in several studies based in Europe, North America and Japan. This variant is also seen in some haplotypes positive for rs1799853 (CYP2C9*2 allele). However, there is no evidence that the variant at position −1188 is associated with altered transcription or warfarin dose requirement [86,112,113]. A TG deletion at position −2663 (rs71486745) that lies within a putative binding site for the transcription factor Nrf2 is relatively common in individuals negative for coding region polymorphisms [86,112,113,114]. This variant is in linkage disequilibrium with the −1188 variant mentioned above and a variant at position −3089 (rs12782374) which lies within another putative transcription factor binding site, this time for YY1 [114]. Similar to the findings for the −1188 variant alone, this haplotype did not affect warfarin or phenytoin dose requirement [86,112,113,114], but in vitro transcription studies indicated that reporter gene constructs including this haplotype may be associated with decreased responsiveness to both rifampicin and phenytoin when PXR and CAR, respectively, are also co-transfected [113,114]. The lower CAR responsiveness was also localized to the −3089 position [114]. This polymorphism, which is seen at a frequency of 0.17 in white Europeans, may be of limited relevance in terms of initial drug dosing, but could affect susceptibility to some DDIs.

Further upstream, a C>T at position −4302 (rs12251841) was detected in Mexicans but not in non-Latino white Americans [113]. The variant appeared to be associated with lower constitutive expression of the promoter region. A further study on phenotyped human livers and patients treated with warfarin detected a variable number of tandem repeats (VNTR) sequence at position −3979 approx. Three patterns that were termed short, medium and long were identified. The medium repeat pattern was the most common and taken as the reference sequence [115]. The short allele appeared to be associated with decreased levels of CYP2C9 transcription in the liver, based on allelic imbalance studies and reporter gene assays. In addition, homozygosity for the short allele was associated with a lower warfarin dose but the overall effect on dose of this variant was less than that seen with CYP2C9*2 and *3 and was not significant in a multiple regression model [115].

Several upstream variants that are part of a haplotype that also includes rs1057910, which codes for the missense mutation in the CYP2C9*3 allele, have also been detected [116]. These variants were originally thought to be associated directly with decreased CYP2C9 activity. As discussed elsewhere [86,112], there is decreased transcriptional activity in reporter gene studies [113,116] but this decrease is on the order of 50% whereas the effect of the Ile359Leu amino acid substitution is larger in terms of overall effect on enzyme activity.

As discussed above, the overall enzyme activity associated with the CYP2C9*8 allele is still not very clear, with in silico predictions suggesting no effect of the Arg150His substitution on enzyme activity, in vitro studies producing contradictory findings, and in vivo studies suggesting decreased activity. An upstream polymorphism −1766 T>C shows strong linkage disequilibrium with rs7900194 in African-Americans and this haplotype also includes the −1188 T>C variant discussed above. Allele imbalance and luciferase reporter gene studies indicate that a −1188C–1766C sequence results in lower levels of transcription compared with the −1188T–1766T sequence [117]. Polymorphisms in the upstream sequence may, therefore, be a better explanation of the apparent decreased in vivo CYP2C9 activity in individuals positive for CYP2C9*8 than the missense mutation. 

A sequencing study on CYP2C9 variation in relation to warfarin dose requirement in African-Americans reported that a variant in intron 3 (rs7089580) was associated with a high warfarin dose requirement. This variant was in linkage disequilibrium with several other intronic variants and it was suggested that one of these might be within a transcription factor binding site [118]. In a genome-wide association study (GWAS) on warfarin dosing in African-Americans, an upstream *CYP2C9* variant rs12777823 exhibited a significant association with dose requirement in addition to CYP2C9*2 and CYP2C9*3 [117]. This variant is upstream of *CYP2C18*, which is located quite a long distance from *CYP2C9* on chromosome 10. As discussed below, there is extensive long-range linkage disequilibrium within the *CYP2C* gene cluster and this variant may be associated with other polymorphisms closer to *CYP2C9*.

### 5.5. Linkage Disequilibrium with Other CYP2C Genes

*CYP2C9* is part of the four-gene *CYP2C* cluster on chromosome 10. *CYP2C9* is flanked upstream by *CYP2C19* and downstream by *CYP2C9* with *CYP2C18* further upstream of *CYP2C19*. A number of studies have investigated linkage disequilibrium between common SNPs in *CYP2C8* and *CYP2C9* with more limited investigation of relationships with *CYP2C19*. Linkage disequilibrium between *CYP2C9* and *CYP2C8* was first reported in 2002 [119] when it was shown that almost all individuals positive for CYP2C9*2 were also positive for CYP2C8*3, an allele with two nonsynonymous mutations that is believed to be associated with decreased activity. In a more detailed study involving a range of different populations [120], it was reported that 90% of European and South West Asian alleles that are positive for the CYP2C9*2-associated Cys144 are also positive for the two CYP2C8*3-associated amino acid substitutions. Individuals positive for Cys144, but negative for the two CYP2C8*3 variants, are seen occasionally in populations where CYP2C9*2 is common (Table 2) but the frequency of this haplotype is low in the populations examined [120]. In view of the overlap in substrate specificity between these two P450s with certain substrates such as ibuprofen and arachidonic acid, this finding is of considerable interest in terms of potential clinical impact.

The relationship between *CYP2C9* and *CYP2C19* genotypes has been examined in a single study [121]. This study confirmed the existence of a number of rare *CYP2C9* variants in Japanese subjects (Table 1), and also found that two upstream *CYP2C9* haplotypes (Section 5.4) were in linkage disequilibrium with the loss of function CYP2C19*2 and CYP2C19*3 alleles respectively. These upstream *CYP2C9* variants are believed not to affect *CYP2C9* expression so it is unlikely that there will be any joint impact on drug metabolism. However, these findings show that linkage disequilibrium within the *CYP2C* region extends from *CYP2C9* upstream to *CYP2C19* and the possibility that impaired function for both enzymes could be seen in some ethnic groups. As discussed in Section 5.4, a polymorphism in *CYP2C18* that is further upstream of *CYP2C9* than *CYP2C19* appears to affect warfarin dose requirement in African-Americans [117].

## 6. Clinical Significance of *CYP2C9* Polymorphisms

### 6.1. Coumarin Anticoagulants

In terms of clinical significance of *CYP2C9* polymorphisms, the most well studied example is the relationship between warfarin dosing and genotype. There are also a number of studies concerned with other coumarin anticoagulants. As discussed in Section 4, *CYP2C9* is the major P450 that contributes to hydroxylation of the key active enantiomers for these compounds. Initial studies showing an effect of *CYP2C9* genotype on warfarin dose involved studies on patients requiring unusually low doses [81,82], but this effect was confirmed subsequently in studies on patients of European ethnicity taking a range of warfarin doses [122,123,124,125]. A significant contribution of the CYP2C9*2 and *3 variants to dose requirement has also been shown by several GWAS [126,127,128,129,130]. A meta-analysis of studies involving multiple ethnicities showed significant effects for CYP2C9*2 on dose requirements in European and African-American populations with all ethnic groups studied to date demonstrating an effect for CYP2C9*3 [131]. Bleeding events due to warfarin have also been studied in relation to *CYP2C9* genotype and the foregoing meta-analysis found an association only for patients with two copies of CYP2C9*3 [131]. The overall contribution of CYP2C9 to warfarin dose requirement is considered in more detail in Section 7, which considers dosing algorithms and contributions by other genetic and non-genetic factors.

For acenocoumarol and phenprocoumon, data on the relationship between *CYP2C9* genotype and dose requirement is more limited compared with warfarin. Though there are reports that *CYP2C9* genotype is less important as a determinant for phenprocoumon dosing than warfarin or acenocoumarol [132], a large study which studied both acenocoumarol and phenprocoumon dosing in parallel reported that *CYP2C9* genotype explained 4.5% of dose variation for acenocoumarol and 4.6% for phenprocoumon [133]. This CYP2C9 contribution to dose requirement is lower than most reports for warfarin. Algorithms incorporating the *CYP2C9* data for phenprocoumon and acenocoumarol dosing were developed and used in a randomised controlled trial which failed to show significant benefit for genotype-guided dosing [134] with these drugs, but generally data on the importance of *CYP2C9* genotype to non-warfarin coumarin anticoagulant treatment remains sparse.

### 6.2. Sulfonylureas

Two large studies have evaluated the relevance of *CYP2C9* genotype to response to sulfonylurea treatment. The earlier of these was concerned with tolbutamide and reported that CYP2C9*3 carriers were prescribed significantly lower doses of this drug than homozygous wild-type patients [135]. A second reported a better response to treatment with a number of different agents, in those carrying CYP2C9*2 or *3 alleles though the majority of these patients were prescribed glicazide and none were taking tolbutamide [136]. There are also limited reports that individuals carrying *CYP2C9* variant alleles are at increased risk of hypoglycaemia [137,138,139].

### 6.3. Nonsteroidal Antiinflammatory Drugs

Unlike the examples in the two previous sections, response to NSAIDs is less easily measured and the majority of studies on CYP2C9 pharmacogenomics in relation to this drug class are concerned with adverse events. One study did investigate efficacy in the context of using celeboxib to prevent colorectal adenoma and found carriage of CYP2C9*3, but not CYP2C9*2, was associated with increased protection against adenoma in those taking high doses though the overall effect was small [140]. For adverse events, the main issues that have been investigated are gastrointestinal bleeding, hepatotoxicity and susceptibility to cardiovascular events. The overall contribution of CYP2C9 to the metabolism of drugs in this class varies and its contribution to clearance of a number of agents, especially those implicated in cardiovascular events, is likely to be insufficient to show important pharmacogenomic associations [141]. However, a number of NSAIDs including ibuprofen, flurbiprofen and celecoxib may be sufficiently CYP2C9-dependent in their clearance for genotype to be relevant. There are several reports suggesting that carriage of *CYP2C9* variants increases the risk of gastrointestinal bleeding to NSAIDs generally [142,143,144] with the highest risk relating to CYP2C9*3. However, individual NSAIDs have not been investigated in a systematic manner. Several NSAIDs are associated with drug-induced liver injury and the best studied member of the class in this context is diclofenac. An increased frequency of *CYP2C9* variant alleles was not detected in diclofenac-induced liver injury cases [145], either genotyping directly for CYP2C9*2 and *3 or in a larger GWAS [146].

### 6.4. Phenytoin

Similar to warfarin, phenytoin has a narrow therapeutic index with pharmacokinetic variability due to *CYP2C9* polymorphisms well established for both CYP2C9*2 and CYP2C9*3 [147]. Effects on the central nervous system are the most common symptom of high plasma drug levels but there is also an increased risk of serious skin rash [148]. Data on the relevance of *CYP2C9* genotype to neurological toxicity are limited but there is a report of such toxicity in a patient with a homozygous CYP2C9*2 genotype [149]. There is also a risk of serious skin rash with phenytoin [148]. The genetic basis of this adverse reaction has been investigated by GWAS in an Asian population and a genome-wide significant signal with CYP2C9*3 reported [150]. Hepatotoxicity reactions are also seen occasionally with phenytoin [151], but the genetic basis for these remains unclear.

### 6.5. Miscellaneous

The relevance of common CYP2C9 polymorphisms to a range of different drug substrates in addition to the major groups discussed above has been investigated. In the case of losartan where both the parent drug and E-3174, the metabolite generated by CYP2C9, are active, the ratio of losartan to E-3174 is genotype-dependent when the effect of CYP2C9*2 and *3 is assessed [152]. There is limited data suggesting that those homozygous or possibly heterozygous for CYP2C9*3 show a poorer anti-hypertensive response [153]. On the other hand, for candesartan and irbesartan where active metabolites are not generated, those positive for CYP2C9 variants are at risk for hypotension [53,154]. 

Bosentan is an endothelin receptor antagonist used for treatment of pulmonary arterial hypertension. There is evidence that CYP2C9 contributes to its oxidative metabolism [155]. It is well established that bosentan can cause drug-induced liver injury and patients taking this drug undergo routine transaminase monitoring as a result [156]. Two recent studies suggest that CYP2C9 variant alleles, particularly CYP2C9*2, increase the risk of developing hepatotoxicity with this drug [157,158]. The effect sizes seen are modest and further work is needed, particularly because CYP2C9 is not the only P450 isoform contributing to metabolism [155].

CYP2C9 contributes to arachidonic acid metabolism converting it to EETs [159]. EETs have vasodilatory and antiinflammatory effects that may be relevant to physiological processes including angiogenesis and regulation of vascular tone. Although CYP2C8 and CYP2J2 also carry out these reactions and may have a more important role in EET metabolism extrahepatically, CYP2C9 is also likely to contribute. As described in more detail elsewhere [56,160], a number of case-control candidate gene studies on associations between *CYP2C9* genotypes and susceptibility to coronary heart disease, coronary artery disease, myocardial infarction and hypertension have been performed. To date, these studies have yielded rather contradictory results. GWAS on cardiovascular diseases have so far failed to detect significant signals for CYP2C9, so despite the biological plausibility of EET production being relevant to disease risk and a relatively large number of studies, evidence for a pharmacological effect in vivo is limited.

## 7. Warfarin Dosing Algorithms

Clearly, *CYP2C9* genotype is an important predictor of warfarin dose requirement. However, a number of other factors contribute to dose, including genotype for vitamin K epoxide reductase (*VKORC1*), which encodes the warfarin target, patient age and patient weight or height.

There have been a number of estimates of the contribution of *CYP2C9* genotype to warfarin dose requirement among Europeans with initial results ranging from 6 to 19% [122,161,162]. For other ethnic groups where the range and frequency of CYP2C9 variants is different, the contribution of CYP2C9 is less well understood but, where known, tends to be lower mainly because variants associated with low activity are rarer in most non-European groups. When the other parameters mentioned above are added, typically the percentage of overall variation in dose requirement that can be estimated is in the order of 50%. Other genes in addition to *CYP2C9* and *VKORC1*, such as *CYP4F2*, which contributes to vitamin K metabolism, are also relevant to warfarin dose requirement but their overall contribution is lower, so these are generally less useful as predictors for dosing.

The earliest warfarin dosing algorithms incorporating genetic factors included only *CYP2C9* genotype [122,161]. Once *VKORC1* was also shown to be of relevance to dosing, algorithms incorporating this second genetic factor were developed [163,164,165,166,167,168,169,170,171]. These algorithms were developed and tested on relatively small patient groups, though overall they were not too dissimilar. One of these algorithms was developed using data from a relatively large number of American patients and is web-based (www.warfarindosing.org) [171]. To further improve ability to predict dose requirement and to cover a wider range of ethnicities, a joint project involving a large number of researchers worldwide (International Warfarin Pharmacogenetics Consortium (IWPC)) used clinical and genetic data on 4043 patients treated with warfarin to develop a more definitive warfarin dosing algorithm which was then tested on a replication cohort of 1009 patients [172]. This was an important step forward in developing a clinically useful algorithm for warfarin dosing, but limitations included an over-representation of European patients and the fact that the only genotypes considered were those for common variant alleles in *CYP2C9* and *VKORC1*. It was also uncertain exactly how an algorithm predicting the stable dose of warfarin should be implemented clinically. Further refinements of the IWPC algorithm have subsequently been made. One refinement introduces a “dose-revision algorithm” which involves initiation of treatment based on the IWPC algorithm-calculated dose followed by use of a new algorithm that incorporates genetic factors, INR value and clinical factors on day 4 or 5 of treatment [173]. Another uses an “initiation dose” algorithm derived from the IWPC algorithm, but introduces an additional term to increase the dose on days 1 to 3 of treatment [174]. Few currently available warfarin dosing algorithms include genetic factors other than the common *CYP2C9* and *VKORC1* variants, but modification of existing algorithms to include *CYP4F2* genotype and a range of rarer *CYP2C9* alleles has been suggested to improve stable dose prediction [175,176]. This has also been implemented in the web-based warfarin dosing calculator [177]. 

Though the IWPC algorithm was developed to cover a wide range of ethnic groups, dosing algorithms have also been developed specifically for specific ethnic groups [88,178,179,180,181]. Children occasionally require treatment with coumarin anticoagulants. Two studies suggest that the algorithms developed for adults are not predictive of dose requirements in this group and have proposed alternative algorithms [182,183]. 

Early clinical trials assessed the use of genotype and other patient-related factors to set initial warfarin dose [167,184,185,186,187,188]. In general, these early studies found no advantage for a genotype-determined dose, but power to detect all genetic effects appears to have been limited. One of these studies involved *CYP2C9* genotypes only and found improved time to stable INR and higher percentage of time within therapeutic range in genotype-guided dose cases compared with controls receiving normal treatment [184]. Another study showed that providing information on CYP2C9 and VKORC1 genotype to the prescriber resulted in better outcomes for warfarin treatment. However, genotype data was only available approx. 32 days after the start of treatment, which is an important limitation [185]. 

The first large randomized control trial (RCT) involved 504 cases treated with a pharmacogenetics-guided dose and 1911 controls given standard dosing [189]. This study showed that the pharmacogenetic-guided dosing group had a higher percentage of patients within therapeutic INR range at two time points and fewer patients showing serious adverse events or very low or high INR values.

Outcomes of two further RCTs were reported in 2013. These RCTs, termed EU-PACT and COAG, were based in Europe and the USA respectively and reported different findings [190,191]. EU-PACT found that patients who received a genotype-guided warfarin dose remained in the target therapeutic range for a significantly longer time in the first three months of treatment compared with patients treated with a standardized conventional dosing regimen. COAG reported no improvement in time within therapeutic range in the first 4 weeks of treatment. These conflicting findings may be due to some differences in study protocol and the participating patients. In particular, the dosing algorithms and genotyping assay protocols were different and in addition COAG included a significant number of African-American patients whereas EU-PACT involved white Europeans only. EU-PACT used a modified IWPC initiation loading dose [174] with a further day 4 or 5 modification [173]. COAG used a modified version of the web-based warfarin dosing calculator algorithm [171], which gives the predicted maintenance dose, but ignored the effect of *CYP2C9* in those patients positive for *CYP2C9* variant alleles for the day 1 dose only. The study also applied a day 4 or 5 dose-refinement algorithm [173]. The two studies were different in the treatment of the control arm, with EU-PACT using a standardized approach but COAG using a clinical dosing algorithm that included factors such as age. All patients in the genetics arm of EU-PACT were genotyped before the first dose of warfarin, whereas genotype data was available for only 45% of COAG patients in the genotyping arm at the first dose (though by day 2 this information was available for 94% of patients). As suggested elsewhere [192], due to use of a maintenance dose algorithm to determine initial dose, it is unlikely that steady state was achieved in most COAG patients by day 4. This would result in limited changes in INR and make the dose-refinement algorithm less useful. It has also been suggested that the benefit of genotyping seen in EU-PACT could be due to the dosing protocol used for the control group, which included only limited initial dose loading [193]. Very recently, the outcome of a further RCT, the GIFT study, has been reported [194]. This study used the web-based warfarin dosing calculator algorithm with the CYP4F2 modification discussed above in a predominantly white American population. The genotype-guided dose arm received the calculated dose for 11 days after initiation and showed a better outcome compared with a control group dosed with a clinically guided algorithm in relation to several parameters including INR above four, bleeding and death. The overall findings appear fairly consistent with EU-PACT. Though the study included 1650 patients, they were older than those in EU-PACT and COAG and undergoing arthoplasty. Consequently, they were not necessarily typical of most patients initiating warfarin treatment so there are some limitations. Similar to EU-PACT, GIFT had genotype data available prior to initiation of dosing. 

An important issue emerging from COAG was poorer outcomes in the genotyped African-Americans compared with the control group. This may reflect the fact that *CYP2C9* genotyping was only for the *2 and *3 alleles. As discussed in detail in Section 5.2, other *CYP2C9* alleles such as CYP2C9*5, *6, *8 and *11 are likely to be relevant to dosing in African-Americans. There are also additional VKORC1 variants that may be relevant to dosing [118]. An algorithm specific to African-Americans that includes provision for additional *CYP2C9* and *VKORC1* alleles has been developed and appears to predict dose requirement more accurately than the IWPC algorithm [88]. The web-based warfarin dosing calculator algorithm has also been modified to include the common African-American *CYP2C9* and *VKORC1* variants and this also led to improved dose prediction [177]. Additional modifications that take into account other *CYP2C9* variants seen internationally (see Section 5.2) may be valuable for these algorithms. 

Comparison of the warfarindosing.com and IWPC algorithms suggests that both predict comparable warfarin doses [193]. A recent study found that in patients requiring 7 mg or greater per day, a range of algorithms including those from warfarindosing.org and IWPC under-predicted maintenance dose requirement [195]. The underlying reason for this issue remains unclear, but it has been suggested that it could reflect the complexity of the blood clotting cascade. It is not clear what further modifications to algorithms can be made to improve this issue. Though this limitation may lead to delayed achievement of target INR in a minority of patients, the algorithms seem particularly useful in preventing excessively high INR values during the early stages of anticoagulation in patients with a low maintenance dose requirement. High INR values (>4) are a risk factor for bleeding, particularly in elderly patients [196] and warfarin related-adverse drug reactions of this type are a common cause of hospital admission [197].

Although not all RCTs to date have provided unequivocal evidence that genotyping for *CYP2C9* and *VKORC1* is beneficial prior to dosing with warfarin, support for genotyping has been obtained in a large RCT which compared warfarin and the direct-acting anticoagulant (DOAC) edoxaban [198]. This study did not genotype at initiation of treatment but was able to demonstrate a clear relationship between the presence of *CYP2C9* or *VKORC1* variant alleles and early bleeding. Approximately 5000 patients were included in the warfarin treatment arm enabling this important endpoint to be analyzed directly. With the development of edoxaban and other DOACs, patients needing oral anticoagulants are increasingly being prescribed these drugs in place of warfarin. This may limit the further application of genotyping to warfarin prescribing though, as discussed recently, there is still a need to comprehensively understand the risk-benefits of these new agents [192] and warfarin is still likely to remain a widely-prescribed drug, especially if appropriate dosing algorithms and genotyping strategies can be incorporated in its routine use. 

## 8. Contemporary Translational Efforts

### 8.1. Pre-Emptive Genotyping

Despite substantial efforts from many research teams world-wide, the incorporation of genotype-guided dosing for CYP2C9-related outcomes is far from routine. However, in North America at least, several large teaching hospitals and research institutions (e.g., Vanderbilt, St. Jude, Mayo Clinic) are actively evaluating the impact of prospective genotyping for an array of pharmacogenes, as is evident from other contributions to this Special Issue. Justification for this comes, in part, from the realization that in any given patient population the proportion that possess an ‘actionable pharmacogene’ is very high, exceeding 90% [55,199]. This term has been coined by the Clinical Pharmacogenetics Implementation consortium (CPIC), which has the goal ‘to help clinicians understand how available genetic test results should be used to optimize drug therapy’ [200]. CYP2C9 has been identified by CPIC as a key component of several level A or B drug–gene pairs wherein prescribing action based on pharmacogenetic information is recommended for therapy with phenytoin, warfarin and celecoxib [201].

### 8.2. The Problem of Variants of Uncertain Significance

However, while the discovery of genetic variation in pharmacogenes continues at a substantial pace, aided by advances in DNA sequencing technologies and the investigation of more targeted disease and under-served ethnic populations, there are several well-recognized barriers that need to be overcome to ensure widespread clinical implementation of pharmacogenomics [202]. A key challenge is the provision of clear, unambiguous guidance to health care providers in use of pharmacogenomic information. This is difficult enough for well-studied alleles such as CYP2C9*2 and CYP2C9*3, but the paucity of information on the functional consequences of rarer pharmacogene alleles and diplotypes is a large impediment to providing comprehensive guidelines to the medical community. The true magnitude of these unmet needs becomes clear when one considers the totality of rare variation that will be identified when comprehensive medical genetic screens become the norm [203]. Exome and genome sequencing is already widely employed as a research tool, and the genome Aggregation Database, which includes 123,136 exomes and 15,496 whole genomes [84], has entries for over 400 rare missense and loss-of-function SNPs (minor allele frequency (MAF) < 0.05) in *CYP2C9*, 227 of which were singletons. Undoubtedly, as genome-wide sequencing of *CYP2C9* continues, many more ‘privileged’ SNPs will be identified. This issue is not confined to *CYP2C9*, as a recent study of rare variation in CYP genes illustrates, where 730 novel non-synonymous variants in 12 CYPs were discovered in the exomes of ~6500 individuals [204]. These variants were individually rare, but ~10% of individuals carried at least one potentially deleterious novel variant at one of these 12 loci. These genes also contain previously known rare (MAF < 0.5–1%) variants whose functional consequences remain unclear. These results, obtained from a limited number of individuals relative to the number of patients who will ultimately be genotyped, raise serious questions about how to deal with this avalanche of information for the so-called ‘variants of uncertain significance’ [205]. One might argue that because nearly all identified new coding variation will be found in the heterozygous state, the impact might only be significant for those null or near-complete loss of function allele. However, even moderate functional impact alleles will be important when they present together with common variants like CYP2C9*2 and CYP2C9*3.

## 9. Future Prospects

### 9.1. Computational Approaches

Currently, the best methods for determining the impact of a newly identified coding variant fall into two categories. First, traditional biochemical assays, such as *S*-warfarin and *S*-flurbiprofen metabolite assays can reveal the functional consequences of *CYP2C9* variants, usually by comparison of their in vitro intrinsic clearance parameters, *V*_m_/*K*_m_. However, these assays are limited in scale to at most hundreds of coding variants and so are unable to deal with the massive numbers of variants that will be revealed in the extant human population. Second, numerous algorithms alluded to earlier (e.g., SIFT, PolyPhen etc.) have been used to assign a probable function to missense mutations. These algorithms have the advantage of being scalable such that they can describe the consequences of any CYP2C9 (or other pharmacogene) variant. However, computational approaches are of limited value at present, often producing incorrect or conflicting results. This problem is illustrated by an analysis of the common I359L (CYP2C9*3) polymorphism in *CYP2C9*. CYP2C9*3 encodes arguably the most important deleterious *CYP2C9* variant because of its relatively high MAF (~7% in Caucasians) and the in vitro and in vivo experimental data that demonstrate ~90% loss of function for this allele. However, the non-synonymous amino acid change is extremely conservative and the PolyPhen 2 score is only 0.02, which is predictive of a benign mutation. While more contemporary algorithms offer some predictive improvements [206], what the field needs is a uniform experimental approach that provides an ‘impact score’ that describes the functional consequences of every possible missense variant at every position in each pharmacogene [207]. Such an approach would allow us not only to make predictions about the alleles that have already been observed, but also to generate a look-up table that can be employed to interpret new alleles discovered in the future.

### 9.2. Large-Scale Functional Assays

With recent advances, especially around deep mutational scanning (DMS), large-scale functional annotation of pharmacogenes is a realizable goal. DMS is a relatively new technology that can quantify the effect of hundreds of thousands of variants of a protein of interest in parallel and at low cost [208,209,210]. Rather than assaying individually chosen variants for their functional consequences, this method can measure the activity or stability of each of many variants of a protein in a single experiment. DMS uses a coupled genotype–phenotype system, where a library encoding protein variants is introduced into an appropriate host cell. A selective pressure is applied to this library of variants, altering the frequency of each variant depending on its level of activity or stability. High-throughput DNA sequencing is used to characterize the frequency of each variant throughout the selection, and the change in frequency is used to calculate a functional score. Variants that are depleted after selection have low functional scores whereas variants that are enriched after selection have high functional scores. The result is a large-scale protein functional data set, identifying important positions, as well as activity-enhancing and loss-of-function mutations. Studies are underway to catalog the effects of the 10,290 possible coding-region variants in *CYP2C9* [211]. Successful completion of this goal will provide not only an invaluable resource to guide decision-making at the clinical level, but also elucidate fundamental biochemical relationships between P450 structure, catalysis and enzyme stability.

## Figures and Tables

**Table 1:** Representative examples of drugs for which CYP2C9 is responsible for >25% of metabolic clearance.

Drug Class	Drugs
Anticoagulants	Acenocoumarol, phenprocoumon, *S*-warfarin
Antihypertensives	Irbesartan, losartan
NSAIDs	Celecoxib, diclofenac, etodolac, ibuprofen, indomethacin, lornoxicam, mefenamic acid, suprofen, tenoxicam
Oral hypoglycemic agents	Chlorpropamide, glibenclamide, gliclazide, glimepiride, nateglinide, tolbutamide
Miscellaneous	Bosentan, fluvastatin, mestranol, phenytoin, torsemide

Taken from references [5,6,7,16,17]. NSAIDs: nonsteroidal anti-inflammatory drugs.

**Table 2:** Variant *CYP2C9* alleles and frequencies in different ethnic groups.

SNP	Effect	*Allele	Sequence Change Europeans	Overall Frequency Worldwide	European Frequency	African Frequency	East Asian Frequency	South Asian Frequency	Effect
rs1799853	p.Arg144Cys	*2	c.430C>T	0.0914	0.1268	0.0235	0.0003	0.046	PolyPhen: probably damaging; SIFT:tolerated; other:impaired *S*-warfarin metabolism in vitro [77] and decreased dose in vivo [82]
rs1057910	p.Ile359Leu	*3	c.1075A>C	0.0637	0.0688	0.0126	0.0338	0.1131	PolyPhen: possibly damaging; SIFT: deleterious; other: impaired *S*-warfarin and tolbutamide metabolism in vitro [78,79] and decreased warfarin dose in vivo [82]
rs2256871	p.His251Arg	*9	c.752A>G	0.0067	0.0002	0.0754	0.0001	0.0001	PolyPhen: probably damaging; SIFT: deleterious; other: no effect on phenytoin clearance in vivo [90]
rs7900194	p.Arg150His	*8	c.449G>A	0.0052	0.0003	0.056	0.0001	0.0006	PolyPhen:benign; SIFT:tolerated; other: increased activity towards tolbutamide in vitro [91], decreased activity towards phenytoin in vivo [90], decreased warfarin activity in vitro and in vivo [92]
rs28371685	p.Arg335Trp	*11	c.1003C>T	0.0038	0.0021	0.0214	0.0001	0.0019	PolyPhen: probably damaging; SIFT: deleterious; other: decreased activity towards warfarin in vivo and in vitro [87,91]
rs72558189	p.Arg125His	*14	c.374G>A	0.003	0.0001	>0.0001	0.0001	0.0204	PolyPhen:benign; SIFT:deleterious; other: very low activity in vitro towards tolbutamide and warfarin [93]
rs9332239	p.Pro489Ser	*12	c.1465C>T	0.0019	0.003	0.0006	0	0.0002	PolyPhen: possibly damaging; SIFT: deleterious; other: decreased warfarin dose requirement [88]
rs2837168	p.Asp360Glu	*5	c.1080C>G	0.0012	<0.0001	0.0127	0	0	PolyPhen: probably damaging; SIFT: deleterious; other: decreased activity towards warfarin and diclofenac in vitro [94]; decreased phenytoin clearance in vivo [90]
rs9332131	p.Lys273Arg (fsTer34)	*6	c.818delA	0.0009	<0.0001	0.0105	0	0	Frameshift so inactivating; other: impaired phenytoin clearance in vivo [95]
rs182132442	p.Pro279Thr	*29	c.835C>A	0.0004	0.0005 *	0	0.0016	0.0001	PolyPhen:benign; SIFT:tolerated; other: decreased activity in vitro with tolbutamide [89]; decreased warfarin clearance in vitro [93]
rs72558192	p.Thr299Ala	*52	c.895A>G	0.0002	0	0	0.0035	0	PolyPhen: probably damaging; SIFT: deleterious; other: mutation of known active residue based on crystal structures of flurbiprofen- and warfarin-bound CYP2C9 [96], decreased activity in vitro with tolbutamide [97]
rs72558187	p.Leu90Pro	*13	c.269T>C	0.0001	0	0	0.002	0	PolyPhen:benign; SIFT:tolerated; other: decreased activity in vitro with tolbutamide [97]
rs7900194	p.Arg150Leu	*27	c.449G>T	0.0001	0	0	0.0017	>0.0001	PolyPhen:benign; SIFT:tolerated; other: decreased activity in vitro with tolbutamide [97]; decreased warfarin clearance in vitro [93]

* 0.0011 in Finns; Adapted from www.exac.org. SIFT: Sorting Intolerant From Tolerant.
